# Subjective Vertical Position Allows Prediction of Postural Deterioration in Patients with Parkinson's Disease

**DOI:** 10.1155/2019/1875435

**Published:** 2019-04-02

**Authors:** Kyohei Mikami, Makoto Shiraishi, Tsutomu Kamo

**Affiliations:** ^1^Department of Rehabilitation, Noborito Neurology Clinic, Kawasaki, Kanagawa, Japan; ^2^Department of Neurology, St. Marianna University School of Medicine, Kawasaki, Kanagawa, Japan; ^3^Department of Neurology, Noborito Neurology Clinic, Kawasaki, Kanagawa, Japan

## Abstract

**Background:**

We believe that, in patients with Parkinson's disease (PD), a forward-directed increase in the subjective vertical position (SV) leads to prolonged worsening of forward flexion of the trunk (FFT) mainly because the body adjusts to the SV. We conducted a study to clarify the relation between the SV angle, FFT angle, and various other clinical measures by comparing baseline values against values obtained 1 year later.

**Methods:**

A total of 39 PD patients (mean age, 71.9 ± 10.1 years; disease duration, 7.2 ± 5.4 years; modified Hoehn & Yahr (mH&Y) score, 2.6 ± 0.7) were enrolled. The Unified Parkinson's Disease Rating Scale score, Mini-Mental State Examination (MMSE) score, mH&Y score, FFT angle, SV angle, and levodopa-equivalent dose (LED) were assessed at the time of enrollment (baseline evaluation) and 1 year later.

**Results:**

Eighteen patients (46%) complied with the protocol and completed the study. Significant increases were observed in the 1-year SV angle (*p*=0.02), MMSE score (*p*=0.008), and LED (*p*=0.001) compared to baseline values. Correlation was observed between the baseline SV angle and baseline and 1-year FFT angles (*r*=0.64,  *p*=0.008 and *r*=0.58,  *p*=0.012, respectively) and between the 1-year SV angle and 1-year FFT angle (*r*=0.63,  *p*=0.005).

**Conclusion:**

Our data suggest that the SV contributes to increased FFT.

## 1. Introduction

As a common clinical symptom in patients with Parkinson's disease (PD), forward flexion of the trunk (FFT) becomes more severe as the disease severity increases [1, 2], and it is an important factor in gait disorder, decreased quality of life, and patients' inability to perform activities of daily living [3–5]. However, FFT is resistant to anti-Parkinson drugs [6, 7], and although the efficacies of botulinum toxin [8, 9], deep brain stimulation [10, 11], and rehabilitation [12, 13] are being tested, effective treatment remains elusive. This difficulty stems from the lack of a clear definition of the disease state [14] due to its multifactorial etiology, with contributing factors that include rigidity [15], dystonia [16], drug inducement [17], and proprioceptive integration [18] and vestibular sensation deficits [19]. On investigating the relationship between FFT and PD patients' subjective vertical position (SV) [20], we found larger SV angles among patients with larger FFT angles. Conversely, large SV angles were also observed in some PD patients with mild or moderate FFT, indicating that the SV angle is not determined by severity of the FFT. On the basis of these findings, we hypothesized that a forward-directed increase in the SV is a factor in long-term exacerbation of FFT. The present study aimed to compare SV and FFT angles obtained upon patients' enrollment (baseline measurements) and 1 year later to clarify the relationship between SV and various clinical markers and increase in the FFT angle at 1 year.

## 2. Methods

### 2.1. Study Patients

A total of 39 individuals with PD (17 men, 22 women aged 71.9 ± 10.1 years) who were outpatients at our facility and met the selection criteria were enrolled in the study. These patients were selected from among 99 outpatients with PD. Inclusion criteria comprised (1) a diagnosis of clinically probable or clinically established PD (according to the International Parkinson and Movement Disorder Society Diagnostic Criteria, 2015 [21]); (2) regular follow-up examinations; (3) consent to and participation in PD assessment during routine care; (4) stages I–IV on the modified Hoehn & Yahr (mH&Y) Scale; (5) confirmation of the ON state at the time of observation; (6) a Mini-Mental State Examination (MMSE) score ≥24; (7) no predictable wearing off lasting for more than half of the day and no sudden, unpredictable wearing off (Unified Parkinson's Disease Rating Scale (UPDRS) Item 39 score ≤2); (8) no psychiatric symptoms, such as severe hallucinations or delusions, that inhibit normal daily living activities (UPDRS Item 2 score <3); (9) ability to maintain a standing position; and (10) range of motion (ROM) in trunk extension ≥5°. Patients in whom PD symptoms, including postural abnormality, exacerbated markedly within 1 week were excluded.

The postural and clinical assessments applied in the present study were those conducted for all patients undergoing rehabilitation therapy at our facility and are aimed at evaluating the effectiveness of the therapy. The therapy included a general exercise regimen for PD patients comprising 15 minutes of aerobic exercise and 60 minutes of muscle stretching and strengthening exercises performed once a week to once every 2 weeks. Written informed consent was obtained from patients who met the inclusion criteria based on thorough verbal and written explanations of the study objective and use of the data. Use of the data was approved by the Ethics Committee of the Japan Primary Care Association (2017-006).

### 2.2. Postural Assessment Items

**Figure 1:**
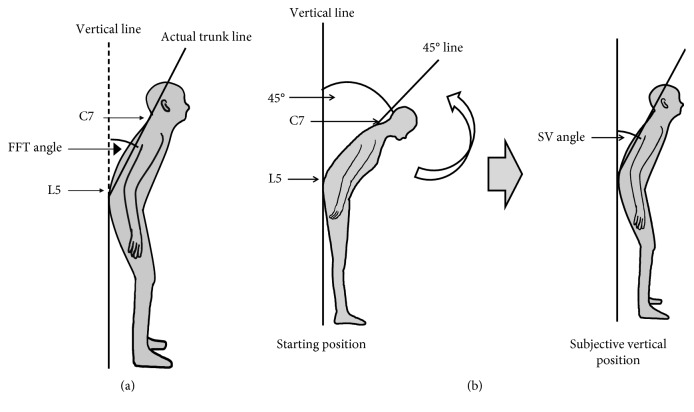
Diagrams illustrating measurement of (a) forward flexion of the trunk (FTT) and (b) subjective vertical position (SV).

Posture was assessed by measuring FFT and SV angles with the use of free image analysis software (Image J; https://imagej.nih.gov/ij/index.html). Still lateral-view images of the patient (90°) were obtained at the level of the iliac crest with the use of a 10.1-megapixel digital camera (Panasonic DMC-LZ10) positioned at a distance of 2 m. On the basis of the Japanese Association of Rehabilitation Medicine's joint ROM measurement method, reflective markers were attached to show the positions of the seventh cervical (C7) and fifth lumbar (L5) spinous processes as analysis landmarks. The FTT angle is determined as follows: the patients stand with eyes open, and the angle between the vertical axis, defined as the line passing vertically through L5 to the floor, and the forward flexion axis, defined as the line connecting C7 and L5 (Figure 1(a)) is measured. The SV angle is then measured as follows: the patient stands with eyes closed but bending forward at the waist so that there is a 45-degree angle between the vertical line drawn on the wall behind the patient and the patient's spine at C7 (Figure 1(b), left panel). The patient's upper body is then passively moved from 45° to 0° in 5 seconds, and the patient is asked to signal when perceiving that he/she has reached a vertical position (Figure 1(b), right panel). At this point, movement is stopped, and a still image is taken. The procedure is repeated three times, the SV angle is measured three times, and the mean value is taken as the patient's SV angle.

### 2.3. Clinical Assessment Items

Data including patients' age, disease duration, and anti-Parkinson drug dosage, including both levodopa-equivalent dose (LED) and use of dopamine agonists, were recorded [22]. Severity of the PD was assessed according to the mH&Y Scale and UPDRS (UPDRS total score). In addition, UPDRS parts I, II, III, and IV (UPDRS I–IV) were used for assessment of cognitive function, activities of daily living, motor function, and treatment complications, respectively.

### 2.4. Statistical Analyses

Data are presented as mean ± standard deviation. Comparisons between baseline and 1-year assessment items were performed by means of the Wilcoxon signed-rank test. Receiver operating characteristic (ROC) analysis was used to test the ability of items showing a significant increase at 1 year to accurately predict increase in the FFT angle at 1 year.

Relations between baseline assessment items, between 1-year assessment items, and between baseline assessment items and the 1-year FFT angle were evaluated on the basis of Spearman's rank correlation coefficient. Multiple regression analysis was performed to investigate the relationship between baseline assessment items and 1-year SV angle and 1-year FFT angle, with baseline FFT angle, baseline SV angle, age, and disease duration used as independent variables. All statistical analyses were performed with SPSS version 21 statistical software (IBM SPSS Statistics for Windows; IBM Corp, Armonk, NY), and significance was set at *p* < 0.05.

## 3. Results

### 3.1. Patient Characteristics

In total, 18 patients (9 men, 9 women; age, 70.3 ± 6.7 years; disease duration, 8.6 ± 6.6 years) recruited between September and October 2015 completed the study, having undergone assessments at the time of enrollment and 1 year later (Table 1). The 1-year assessments could not be performed in 21 patients because of a change in the primary doctor due to hospitalization, admission to a residential facility, or a similar circumstance (*n*=10); difficulty maintaining a standing position due to motor impairment (*n*=5); ROM in trunk extension ≤5° due to low back pain (*n*=4); or difficulty understanding instructions due to mental or cognitive impairment (*n*=2).

### 3.2. Relations between Baseline Assessment Items

The baseline variables did not differ significantly between the 18 patients who completed the study and 21 patients who did not complete the study (Table 2). Among patients who did complete the study, significant correlation was found between the following baseline assessment items: FFT angle and SV angle (*r*=0.58,  *p*=0.012); mH&Y score and disease duration (*r*=−0.51,  *p*=0.03); UPDRS II score and UPDRS III score (*r*=0.77,  *p* < 0.0001) and UPDRS total score (*r*=0.89,  *p* < 0.0001); and UPDRS III score and UPDRS IV score (*r*=−0.48,  *p*=0.045) and UPDRS total score (*r*=9.1,  *p* < 0.0001). No significant correlation was observed between age, MMSE score, UPDRS I score, or LED and any of the other items assessed (Table 3).

### 3.3. Changes in Assessment Items at 1 Year and Results of Correlation Analysis

Changes in assessment items at 1 year for the 18 patients who were included in the study are shown in Table 4. Compared to baseline values, significant increases were observed in 1-year SV angle (*p*=0.02), MMSE score (*p*=0.008), and LED (*p*=0.001). No difference was observed in the mH&Y score, UPDRS III score, UPDRS total score, FFT angle, or dopamine agonist use. Changes in 1-year SV angle, MMSE score, and LED did not correlate significantly with disease duration. The area under the ROC curve showed the prediction accuracies of the baseline MMSE score, SV angle, and LED for an increase in the 1-year FFT angle to be 0.400 (95% confidence interval (CI), 0.122–0.678), 0.377 (95% CI, 0.039–0715), and 0.677 (95% CI, 0.432–0.922), respectively.

Significant correlation was observed for the following 1-year assessment items: FFT angle with SV angle (*r*=0.64,  *p*=0.008) and UPDRS I score (*r*=−0.53,  *p*=0.024); UPDRS I score with UPDRS II (*r*=0.64,  *p*=0.004), UPDRS III (*r*=0.56,  *p*=0.016), and UPDRS total (*r*=0.69,  *p*=0.001) scores; UPDRS II score with UPDRS III (*r*=0.62,  *p*=0.006) and UPDRS total (*r*=0.82,  *p* < 0.001) scores; UPDRS III score with UPDRS total score (*r*=0.93,  *p* < 0.001); and UPDRS IV score with disease duration (*r*=0.72,  *p*=0.001). No significant correlation was observed between age, MMSE score, mH&Y score, or LED and any of the other 1-year assessment items (Table 5).

### 3.4. Relationships between Baseline and 1-Year Follow-Up Assessment Items

**Figure 2:**
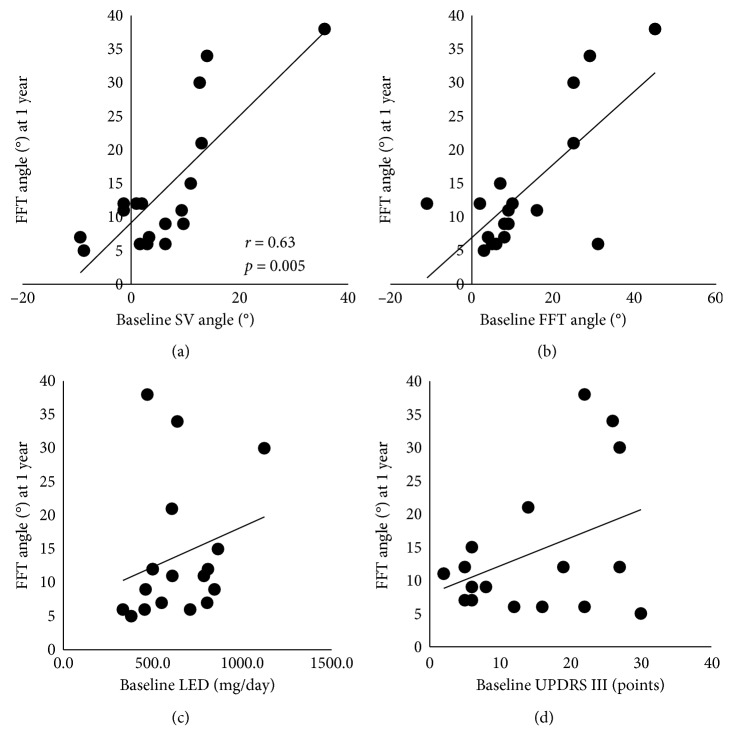
Regression lines showing the relationships between the 1-year FFT angle and (a) baseline SV angle, (b) baseline FFT angle, (c) baseline LED, and (d) baseline UPDRS III score (D). SV, subjective vertical position; FFT, forward flexion of trunk; LED, levodopa-equivalent dose; UPDRS, Unified Parkinson's Disease Rating Scale.

The SV angle was the only baseline assessment item that correlated significantly with the 1-year FFT angle (*r* = 0.63, *p* = 0.005; Figure 2(a)). No significant correlation was observed between the 1-year FFT angle and the baseline FFT angle (Figure 2(b)), baseline LED (Figure 2(c)), baseline UPDRS part III score (Figure 2(d)), age, disease duration, mH&Y score, MMSE score, UPDRS I–II and IV scores, or UPDRS total score. In addition, no significant correlation was found between change in the SV angle (4.9 ± 7.9) and change in the FFT angle (1.1 ± 9.0) at 1 year (*r*=0.29).

## 4. Discussion

On the basis of the hypothesis that a forward-directed increase in the SV is an exacerbating factor for FFT in patients with PD, changes in clinical markers, including the SV angle and FFT angle, were investigated after 1 year. Positive correlation was observed between the SV angle and the FFT angle, both at baseline and 1 year later, indicating a relationship between SV and FFT, as previously reported [20]. No significant change was found in the FTT angle after 1 year, and no significant correlation was found between the baseline and 1-year FFT angles, indicating that the baseline FFT angle is not a contributing factor to the change in FFT angle at 1 year.

Generally, when a patient's bending posture worsens, the patient's disease status has deteriorated, as evidenced by an increase in other signs and symptoms, but, notably, FTT has been shown not to worsen markedly in patients who maintain a high level of physical activity [23]. Our study patients' UPDRS and mH&Y scores did not change significantly, suggesting that their motor function deteriorated only mildly during the observation period. These patients were undergoing rehabilitation therapy regularly, and this might have contributed to what turned out to be a rather modest change in FTT. Although rehabilitation therapy has been reported to positively affect FFT [24, 25], no such effect on SV (either from physical activity or rehabilitation therapy) has been reported. The SV of PD patients may worsen as a result of deterioration of patients' intrinsic sense and sensory integration [18, 26, 27] or of abnormal multisensory integration [28, 29] in the basal ganglia. That is, in the case of FFT and SV, it is argued that the difference in the degree of exacerbation of the FFT and SV distortion is reflected in the difference in change in the measured values. As a result, correlation between the SV angle and FTT angle persists, and SV distortion occurs prior to a negative change in FFT and may signal exacerbation of FFT. In addition, increased SV distortion along with increased FFT is thought to parallel prolonged physical inactivity in the absence of rehabilitation therapy. Multiple logistic regression analysis showed the SV angle to be a factor contributing to the 1-year FFT angle. The present findings support our previously reported findings that patients with a large FFT angle also have a large SV angle and also support our hypothesis that the SV distortion is an exacerbating factor for FFT [20]. Although no previous studies have directly investigated the effects of SV on FFT, Vaugoyeau et al. reported that proprioceptive integration deficits contribute to PD patients' difficulty in maintaining a vertical position [18].

Not only a significant increase in the SV angle but also a significant 1.4-point decrease in the MMSE score was observed after 1 year. The prevalence of cognitive impairment after 10 and 20 years of PD is reported to be 46% [30] and 83% [31], respectively. The MMSE is used in PD patients to screen for cognitive impairment rather than to show specific cognitive deficits or executive dysfunction [32]. Although patients' MMSE scores decreased mildly by 1.4 points along with the change in posture, we found no correlation between patients' MMSE scores and the SV angle or FFT angle, and thus the MMSE score cannot be used as an index to assess posture abnormality. Cognitive function based on somatic [18] and vestibular [19] sensation reportedly plays a role in perception of the vertical position of the body [33]. Thus, perception of the vertical position as represented by the SV angle relies on cognitive function based on processing of sensory information rather than that assessed by the MMSE. Broader assessment of cognitive is needed to clarify the kinds of cognitive function that affect perception of the vertical position of the body as represented by the SV angle in PD patients. In this study, the patients' LED was significantly increased at 1 year, but because FFT did not worsen significantly and actually improved in some patients, we need to further verify the significance of SV.

The limitations of the present study include the small patient group (*n*=18). Furthermore, patients with severe PD, such as those with mH&Y stage V disease, with an MMSE score <24, with severe psychiatric symptoms such as hallucinations or delusions, or with severe wearing off, were excluded; therefore, further investigation is required to determine the effects of increasing symptom severity on FFT. No countermeasure has been developed to prevent alterations in the SV. Such a countermeasure is needed, and we believe that FFT should be evaluated in patients yearly, with a view toward maintenance of the SV. Finally, the influence of rehabilitation therapy could not be ruled out because there was no significant change between the baseline and 1-year follow-up measures of FFT.

## Figures and Tables

**Table 1:** Characteristics of the study patients (*n*=18).

Age (*y*)	70.3 ± 6.7
Sex (male/female)	9/9
Disease duration (years)	8.6 ± 6.6
mH&Y score	2.5 ± 0.6
MMSE score	28.3 ± 1.7
FFT angle (°)	12.8 ± 12.9
SV angle (°)	6.0 ± 9.8
UPDRS part III	14.2 ± 9.4.
UPDRS total	27.3 ± 13.7
LED (mg/day)	636 ± 199.4

Mean ± SD values or numbers of patients are shown. mH&Y, modified Hoehn & Yahr Scale; MMSE, Mini-Mental State Examination; FFT angle, forward flexion of trunk angle; SV angle, subjective vertical position angle; UPDRS, Unified Parkinson's Disease Rating Scale; LED, levodopa-equivalent dose.

**Table 2:** Baseline characteristics of patients who underwent 1 year of follow-up and those who did not.

	Patients who were followed up for 1 year (*n*=18)	Patients who were not followed up for 1 year (*n*=21)	*p* value
Age (years)	70.3 ± 6.7	73.2 ± 11.6	0.35
Sex (male/female)	9/9	8/13	0.45
Disease duration (years)	8.6 ± 6.6	6.0 ± 3.5	0.28
MMSE	28.3 ± 1.7	27.3 ± 2.2	0.22
mH&Y	2.5 ± 0.6	2.6 ± 0.8	0.90
FFT angle (°)	12.8 ± 12.9	8.0 ± 15.0	0.23
SV angle (°)	6.0 ± 9.8	4.5 ± 9.9	0.77
UPDRS I	2.2 ± 1.6	2.0 ± 1.2	0.92
UPDRS II	8.6 ± 6.0	7.3 ± 5.2	0.64
UPDRS III	14.2 ± 9.4	16.2 ± 8.9	0.50
UPDRS IV	2.4 ± 2.8	2.4 ± 2.2	0.69
UPDRS total	27.3 ± 13.7	28.0 ± 13.9	0.88
LED (mg/day)	636 ± 199.4	749.0 ± 349.5	0.25

MMSE, Mini-Mental State Examination; mH&Y, modified Hoehn & Yahr Scale; FFT, forward flexion of trunk; SV, subjective vertical position; UPDRS, Unified Parkinson's Disease Rating Scale; LED, levodopa-equivalent dose.

**Table 3:** Baseline variables and relationships between them.

	Mean	Range	Age (years)	Disease duration (years)	MMSE	mH&Y	FFT angle (°)	SV angle (°)	UPDRS I	UPDRS II	UPDRS III	UPDRS IV	UPDRS total	LED (mg/day)
Age (years)	70.3	55–83												
Disease duration (years)	8.6	1–26	−0.428											
MMSE score	28.3	25–30	−0.386	0.074										
mH&Y score	2.5	1–3.5	0.119	**−0.508** ^*∗*^	0.081									
FFT angle (°)	12.8	−11–45	−0.018	0.343	−0.125	−0.404								
SV angle (°)	6.0	−9.3–35.7	−0.187	0.219	0.177	−0.096	**0.580** ^*∗*^							
UPDRS I	2.2	0–5	0.217	0.068	0.088	0.127	−0.007	0.227						
UPDRS II	8.6	1–20	0.250	−0.125	−0.094	−0.136	0.264	0.351	0.292					
UPDRS III	14.2	2–30	0.338	−0.373	−0.309	−0.083	0.032	0.171	0.139	**0.771** ^*∗*^				
UPDRS IV	2.4	0–11	−0.221	0.449	0.122	0.138	−0.169	−0.236	−0.412	−0.334	**−0.478** ^*∗*^			
UPDRS total	27.3	9–50	0.260	−0.091	−0.292	−0.208	0.142	0.214	0.254	**0.889** ^*∗*^	**0.914** ^*∗*^	−0.293		
LED (mg/day)	635	333–1124	−0.297	0.233	0.085	−0.243	−0.075	0.149	−0.276	0.160	−0.065	0.253	0.034	

Values shown are Spearman correlation coefficients, unless otherwise indicated. MMSE, Mini-Mental State Examination; mH&Y Scale, modified Hoehn & Yahr Scale; FFT angle, forward flexion of trunk angle; SV angle, subjective vertical position angle; UPDRS, Unified Parkinson's Disease Rating Scale; LED, levodopa-equivalent dose. ^*∗*^*p* < 0.05.

**Table 4:** Clinical assessment values upon enrollment (baseline) and after 1 year of follow-up.

	Baseline	1 year	*p* value
mH&Y score	2.5 ± 0.6	2.5 ± 0.7	0.7
UPDRS I	2.2 ± 1.6	2.4 ± 1.7	0.7
UPDRS II	8.6 ± 6.0	6.7 ± 3.9	0.3
UPDRS III	14.2 ± 9.4	16.5 ± 9.9	0.4
UPDRS IV	2.4 ± 2.8	2.2 ± 2.1	0.7
UPDRS total	27.3 ± 13.7	27.8 ± 13.8	0.9
MMSE	28.3 ± 1.7	26.9 ± 2.3	0.008
FFT angle (°)	12.8 ± 12.9	13.9 ± 9.8	0.6
SV angle (°)	6.0 ± 9.8	11.0 ± 6.1	0.02
LED (mg/day)	636 ± 199.4	939 ± 368.4	0.001
Dopamine agonist (%)	100	94.4	0.5

Mean ± SD values are shown. mH&Y Scale, modified Hoehn & Yahr Scale; MMSE, Mini-Mental State Examination; FFT angle, forward flexion of trunk angle; SV angle, subjective vertical position angle; UPDRS, Unified Parkinson's Disease Rating Scale; LED, levodopa-equivalent dose.

**Table 5:** Relationships between study variables after 1 year of follow-up.

	Mean	Range	Age (years)	Disease duration (years)	MMSE	mH&Y	FFT angle (°)	SV angle (°)	UPDRS I	UPDRS II	UPDRS III	UPDRS IV	UPDRS total	LED (mg/day)
Age (years)	71.3	56–84												
Disease duration (years)	9.8	2–28	−0.449											
MMSE	26.9	21–30	−0.286	−0.149										
mH&Y	2.5	1–4	−0.101	−0.414	0.223									
FFT angle (°)	13.9	5–38	−0.227	0.218	0.114	−0.181								
SV angle (°)	11.0	3–23	−0.359	0.139	−0.012	0.198	**0.639** ^*∗*^							
UPDRS I	2.4	0–5	−0.057	0.113	−0.296	0.237	**−0.529** ^*∗*^	0.060						
UPDRS II	6.7	0–14	0.008	0.027	−0.386	0.164	−0.383	0.064	**0.639** ^*∗*^					
UPDRS III	16.5	3–35	0.128	0.057	−0.351	−0.012	−0.270	0.192	**0.561** ^*∗*^	**0.624** ^*∗∗*^				
UPDRS IV	2.2	0–6	−0.223	**0.722** ^*∗*^	−0.151	−0.129	−0.158	−0.199	0.179	0.105	−0.196			
UPDRS total	27.8	7–49	0.012	0.155	−0.397	0.036	−0.381	0.119	**0.692** ^*∗*^	**0.821** ^*∗*^	**0.928** ^*∗*^	0.032		
LED (mg/day)	938	425–1849	0.109	0.366	−0.203	−0.284	0.088	−0.035	−0.163	0.223	−0.103	0.461	0.043	

Values shown are Spearman correlation coefficients, unless otherwise indicated. MMSE, Mini-Mental State Examination; mH&Y Scale, modified Hoehn & Yahr Scale; FFT angle, forward flexion of trunk angle; SV angle, subjective vertical position angle; UPDRS, Unified Parkinson's Disease Rating Scale; LED, levodopa-equivalent dose. ^*∗*^*p* < 0.05.

## Data Availability

The data used to support the findings of this study are included within the article.
